# S2k‐Leitlinie Berufliche Hautmittel: Hautschutz, Hautpflege und Hautreinigung

**DOI:** 10.1111/ddg.70005

**Published:** 2026-05-05

**Authors:** Michal Gina, Manigé Fartasch, Andrea Bauer, Hans Drexler, Peter Elsner, Johannes Geier, Swen Malte John, Sybille Schliemann, Christiane Altenburg, Richard Brans, Dominique Jasmin Lunter, Alexander Nast, Birgit Pieper, Axel Schlieter, Petra Staubach, Anja Thielitz, Christoph Zeyen

**Affiliations:** ^1^ Institut für Prävention und Arbeitsmedizin der DGUV, Institut der Ruhr‐Universität Bochum (IPA) Referat Berufsdermatologie Bochum Deutschland; ^2^ Klinik und Poliklinik für Dermatologie Universitätsklinikum Carl Gustav Carus Technische Universität Dresden Dresden Deutschland; ^3^ Institut und Poliklinik für Arbeits‐ Sozial‐ und Umweltmedizin Friedrich‐Alexander‐Universität Erlangen‐Nürnberg Erlangen Deutschland; ^4^ Privatpraxis für Dermatologie und Allergologie SRH Wald‐Klinikum Gera Deutschland; ^5^ Informationsverbund Dermatologischer Kliniken (IVDK) Universitätsmedizin Göttingen Göttingen Deutschland; ^6^ Institut für interdisziplinäre Dermatologische Prävention und Rehabilitation (iDerm) an der Universität Osnabrück Osnabrück Deutschland und Abteilung Dermatologie Umweltmedizin Gesundheitstheorie Universität Osnabrück Osnabrück Deutschland; ^7^ Berufsgenossenschaft für Gesundheitsdienst und Wohlfahrtspflege (BGW) Abteilung Arbeitsmedizin Gefahrstoffe und Gesundheitswissenschaften (AGG) Bereich Arbeitsmedizin Hamburg Deutschland; ^8^ Pharmazeutische Technologie Teilbereich Pharmazie Mathematisch‐Naturwissenschaftliche Fakultät der Eberhard Karls Universität Tübingen Tübingen Deutschland; ^9^ Division of Evidence‐Based Medicine (dEBM) Klinik für Dermatologie Venerologie und Allergologie Charité – Universitätsmedizin Berlin corporate member of Freie Universität Berlin and Humboldt‐Universität zu Berlin Berlin Deutschland; ^10^ Sachgebiet Hautschutz Berufsgenossenschaft Holz und Metall (BGHM) Fachbereich Persönliche Schutzausrüstungen Deutsche Gesetzliche Unfallversicherung (DGUV) Dortmund Deutschland; ^11^ Occupational Health BASF Coatings GmbH Münster Deutschland; ^12^ Hautklinik und Poliklinik Universitätsmedizin Mainz Mainz Deutschland; ^13^ Privatpraxis Dermathie Haldensleben Deutschland

**Keywords:** Berufliche Hautmittel, Hautpflegemittel, Hautreinigung, Hautschutzmittel, Prävention, barrier creams/skin protection creams, Occupational skin products, prevention, skin care products, skin cleansing

## Abstract

Die AWMF‐S1‐Leitlinie 013‐056 „Berufliche Hautmittel: Hautschutz, Hautpflege und Hautreinigung“ aus dem Jahr 2014 wurde grundlegend überarbeitet und auf das S2k‐Niveau angehoben. Ziel der Aktualisierung war die Entwicklung einer evidenz‐ und konsensbasierten Entscheidungshilfe für den zielgerichteten Einsatz beruflicher Hautmittel zur Prävention von Handekzemen am Arbeitsplatz.

Die multidisziplinäre Expertenkommission setzte sich aus Vertretern dermatologischer, arbeitsmedizinischer und dermopharmazeutischer Fachgesellschaften sowie der Unfallversicherungsträger zusammen. Die Aktualisierung erfolgte auf Basis einer nicht systematischen Auswertung der aktuellen nationalen und internationalen wissenschaftlichen Literatur mit besonderem Fokus auf randomisierte kontrollierte Studien.

Die überarbeitete Leitlinie integriert: *(1)* epidemiologische Studien zum Nachweis der Wirksamkeit von Hautschutz und ‐pflege, *(2)* Ergebnisse des Cochrane Reviews zur Primärprävention berufsbedingter Handekzeme, *(3)* hautphysiologische Multicenter‐Studien zur Anwendung und zum Nutzen von Hautschutzmittel sowie zur Verträglichkeit von Hautreinigungsprodukten, *(4)* spezifische Untersuchungen zur Feuchtarbeit und *(5)* aktuelle Positionierungen zu Aluminiumchlorhydroxid basierend auf den Stellungnahmen der *Scientific Committee on Consumer Safety* (SCCS) und zu Kontaktallergenen nach den Daten des Informationsverbunds Dermatologischer Kliniken (IVDK).

Die Empfehlungen und Kernaussagen wurden in einer strukturierten Konsensuskonferenz unter professioneller Moderation mittels nominalem Gruppenprozess konsentiert. Die S2K‐Leitlinie soll zur Verbesserung der Prävention berufsbedingter Handekzeme beitragen und die Gesundheit der Beschäftigten nachhaltig fördern.

## EINLEITUNG

Die vorliegende Leitlinie stellt eine Aktualisierung und ein *Upgrade* der bisherigen S1‐AWMF Register‐Nr. 013/056 „Berufliche Hautmittel“ dar, die 2009 von der Arbeitsgemeinschaft für Berufs‐ und Umweltdermatologie e. V. (ABD) der Deutschen Dermatologischen Gesellschaft und Deutscher Gesellschaft für Arbeits‐ und Umweltmedizin (DGAUM) herausgegeben,^1^ und zuletzt 2014 aktualisiert wurde.^2^ Die Leitlinie behandelt Hautschutz‐, Hautpflege‐ und Hautreinigungsmittel (zusammengefasst als berufliche Hautmittel), die den Beschäftigten eines Betriebs im Rahmen eines integrativen Hautschutzkonzepts zur Prävention beruflich bedingter Hauterkrankungen vom Arbeitgeber zur Verfügung gestellt werden.^3^ Berufliche Hautmittel gehören zu den sogenannten persönlichen Schutzmaßnahmen und im juristischen Sinne unterliegen sie als Kosmetika der EU Kosmetikverordnung Kapitel 5.

Die aktuell vorliegende Leitlinie basiert auf dem gegenwärtigen wissenschaftlichen Erkenntnisstand, wie er sich derzeit aus der nationalen und internationalen Literatur ergibt. Das Upgrade erfolgte unter Berücksichtigung der Vorversion und weiterer aktueller europäischer evidenz‐ und konsensbasierter Leitlinien. Zudem erfolgte eine narrative Aufarbeitung der verfügbaren (inter)nationalen wissenschaftlichen Literatur.

Aus einer Vielzahl von Gründen (Förderung der *Compliance*, Kosten‐Nutzen‐Analyse, potenzielle Risiken präventiver Maßnahmen) sollte darauf geachtet werden, dass nur solche präventiven Maßnahmen empfohlen werden, deren Wirksamkeit wissenschaftlich belegt ist. Aktuell sind für berufliche Hautmittel nur teilweise Wirksamkeitsnachweise vorhanden, so dass konsensbasierte Empfehlungen ausgesprochen werden, welche auf Expertenkonsens beruhen. Eine individuelle Überprüfung der Hautmittel hinsichtlich ihrer Wirksamkeit wird aber durch die unter Kapitel 3 genannten Methoden ausdrücklich angestrebt.

Ergänzend zu dieser Leitlinie wird verwiesen auf die gesetzlichen Rahmenbedingungen durch die EU‐Kosmetik‐Verordnung sowie national durch die Technischen Regeln für Gefahrstoffe (TRGS) 401 „Gefährdung durch Hautkontakt, Ermittlung – Beurteilung – Maßnahmen“ veröffentlicht durch das Bundesministerium für Arbeit und Soziales. Ebenso wird verwiesen auf die DGUV‐Information 212‐017 „Auswahl, Bereitstellung und Benutzung von beruflichen Hautmitteln“ der Deutschen Gesetzlichen Unfallversicherung (DGUV).

*Für die Abschnitte „Hinweise zur Anwendung der Leitlinie/Haftungsausschluss“ (diese gelten in gleichem Maße für die vorliegende Kurzfassung), „Finanzierung“, „Geltungsbereich, Anwenderzielgruppe und Ziele der Leitlinie“ sowie „Adressaten/Anwender“ siehe Langfassung*.

### Nomenklatur

Die Kommission dieser Leitlinie hat sich auf den Begriff „Hautmittel“ in dieser Leitlinie verständigt. Dabei handelt es sich um Externa zum Hautschutz sowie zur Hautpflege und Hautreinigung.

### Empfehlungsstärken, Wording und Symbolik

Eine Darstellung der Wortwahl, Symbolik und Hinweise zur Interpretation der Empfehlungsstärken ist in Tabelle 1 dargestellt.

**TABELLE 1:** Empfehlungsstärken – Wortwahl, Symbolik und Interpretation (modifiziert nach Kaminski‐Hartenthaler et al., 2014^4^).

Empfehlungsstärke	Wortwahl	Symbol	Interpretation
*Starke* Empfehlung *für* eine Vorgehensweise	„… soll …”	↑↑	Wir sind der Auffassung, dass alle oder fast alle informierten Menschen diese Entscheidung treffen würden. Klinikern müssen sich weniger Zeit für den Prozess der Entscheidungsfindung mit den Patienten nehmen. In den meisten klinischen Situationen kann die Empfehlung als allgemeine Vorgehensweise übernommen werden.
Empfehlung *für* eine Vorgehensweise	“… sollte …”	↑	Wir sind der Auffassung, dass die meisten informierten Menschen, ein substanzieller Anteil jedoch nicht, diese Entscheidung treffen würden. Klinikern und andere Anbieter von Gesundheitsleistungen müssen mehr Zeit aufwenden, um sicherzustellen, dass die Wahl des Verfahrens mitsamt den möglicherweise verbundenen Konsequenzen die Werte und Präferenzen individueller Patienten widerspiegelt. Entscheidungsprozesse im Gesundheitssystem erfordern eine tiefgehende Diskussion und die Einbeziehung vieler Stakeholder.
*Offene Empfehlung* bezüglich einer Vorgehensweise	„… kann …”	<–>	Zurzeit kann eine Empfehlung für oder gegen eine bestimmte Vorgehensweise aufgrund bestimmter Gegebenheiten nicht getroffen werden (zum Beispiel keine verfügbare Evidenz, unklares oder ungünstiges Nutzen‐/Risiko‐Verhältnis, etc.)
Empfehlung *gegen* eine Vorgehensweise	„… sollte nicht …“	↓	Wir sind der Auffassung, dass die meisten informierten Menschen, ein substanzieller Anteil jedoch nicht, diese Entscheidung treffen würden.
Starke Empfehlung *gegen* eine Vorgehensweise	“… soll nicht …”	↓↓	Wir sind der Auffassung, dass alle oder fast alle informierten Menschen diese Entscheidung treffen würden.

## BERUFLICHE HAUTMITTEL – DEFINITION UND BEDEUTUNG AM ARBEITSPLATZ

Unter beruflichen Hautmitteln werden in dieser Leitlinie Hautschutz‐, Hautpflege‐ und Hautreinigungsprodukte für den beruflichen Einsatz verstanden und zusammengefasst.

Im engeren Sinne werden unter dem Begriff „Hautschutzmittel” äußerlich anzuwendende Produkte verstanden, welche die Haut vor allem vor Irritationen am Arbeitsplatz schützen sollen (Protektion).^5^, ^6^ Geeignete Produkte sind im Rahmen eines Konzeptes zum integrativen Hautschutz (auch als 3‐Säulen‐Modell bezeichnet) am Arbeitsplatz vor Arbeitsbeginn und während der Arbeit anzuwenden; dabei werden Hautschutzmittel ergänzt durch sogenannte milde Hautreinigungsmittel, die Schmutz und aggressive Substanzen schonend von der Haut entfernen sollen, sowie durch Hautpflegemittel, das bedeutet Produkte, die zur Regeneration der epidermalen Hautbarriere (Pflege/Regeneration) beitragen können.^7^, ^8^, ^9^ Aus Sicht der hautschutzmittelherstellenden Industrie werden unter dem Begriff „Hautschutzmittel“ jedoch nicht nur Produkte zur Verminderung potenzieller Irritationen durch Arbeitsstoffe verstanden, sondern auch Produkte, die zum Beispiel eine erleichterte Reinigung der Haut durch die vorherige Anwendung ermöglichen. Da es hierzu bisher keine gesicherten wissenschaftlichen Untersuchungen und Einstufungen gibt, werden diese im Rahmen der Leitlinie nicht abgehandelt.

Manche Zusätze, die typischerweise in Hautpflegemitteln verwendet werden, sind für den Einsatz in Hautschutzmitteln nicht geeignet wie dies zum Beispiel für Harnstoff (Urea) der Fall ist, dessen potenzielle Penetrationsförderung eine Anwendung vor und während der Tätigkeit nicht empfehlenswert macht.

Der überwiegende Anwendungsbereich beruflicher Hautmittel liegt in der Verhinderung irritativer Kontaktekzeme (Synonym: subtoxisch‐kumulatives Kontaktekzem), die gehäuft an Arbeitsplätzen mit repetitiver Exposition gegenüber schwach reizenden Stoffen und durch Feuchtarbeit entstehen.

In hautgefährdenden Berufen treten Handekzeme mit einer Punktprävalenz von bis zu 40 % auf.^10^ In Risikoberufen sollten Hautgefährdungen im Rahmen der Gefährdungsbeurteilung (GBU) evaluiert und adressiert werden. Zum Beispiel sollten je nach Intensität der Feuchtarbeit (im Sinne der TRGS 401) arbeitsmedizinische Vorsorgeuntersuchungen (Verordnung zur arbeitsmedizinischen Vorsorge, ArbMedVV) erfolgen, in denen Maßnahmen der Prävention erläutert werden. Im Rahmen solcher Untersuchungen sollte eine entsprechende Beratung über die hautschonende Arbeitsweise und Nutzung der Hautmittel am Arbeitsplatz erfolgen sowie die Inhalte des Hautschutzplanes erläutert werden, welcher die Anwendung der Hautmittel am Arbeitsplatz festlegt (siehe Kapitel 4).^3^

## WIRKSAMKEIT VON HAUTSCHUTZ‐, HAUTPFLEGE‐ UND HAUTREINIGUNGSMITTELN

Ein aktueller Cochrane‐Review mit Metaanalyse von randomisierten kontrollierten Studien zur Primärprävention von irritativen Handekzem zeigte, dass die regelmäßige Anwendung von Hautpflegeprodukten, allein (Relatives Risiko [RR] 0,71; 95 %‐Konfidenzintervall [KI] 0,46–1,09) oder in Kombination mit Hautschutzmitteln (RR 0,68; 95 %‐KI 0,33–1,42), einen Trend zur Risikoreduktion aufweist, wobei dieser Effekt keine statistische Signifikanz erreichte. Ähnliche Ergebnisse lieferten die Auswertungen gesundheitspädagogischer Interventionen, die ebenfalls nicht statistisch signifikant waren (RR 0.76, 95 %‐KI 0,54–1,08). Die publizierte Evidenz zu Hautschutz‐ und Pflegeprodukten oder gesundheitspädagogischen Interventionen ist in Summe nicht ausreichend, um die Wirksamkeit von Maßnahmen zur Primärprävention sicher beurteilen zu können.^11^

### Wirksamkeitsnachweise von Hautschutzmitteln

Die Wirksamkeit eines Hautschutzmittels kann weder aufgrund einer theoretischen Betrachtung des jeweiligen galenischen Systems, hydrophiler beziehungsweise lipophiler Eigenschaften, noch des pH‐Wertes beurteilt werden. Da randomisierte kontrollierte Studien/Interventionsstudien unter Arbeitsplatzbedingungen aufgrund organisatorischer und methodischer Schwierigkeiten und der benötigten Ressourcen nicht für jedes Präparat und jede spezifische Exposition durchführbar sind,^5^, ^12^ werden zum Wirksamkeitsnachweis Modellexperimente unter idealisierten Bedingungen genutzt. Hierbei wird die Wirksamkeit eines Hautschutzmittels durch die Beurteilung der Minderung eines irritativen Effektes auf der Haut in vivo mittels der visuellen Beurteilung und sehr häufig durch Bioengineering‐Techniken (hautphysiologische Messmethoden) quantifiziert. Die Aussagekraft der Ergebnisse von modellhaften Irritationstests ist jedoch dadurch eingeschränkt, dass es in der Arbeitswelt oft zu einer komplexen Exposition gegenüber einer Vielzahl von sowohl hydrophilen als auch lipophilen Berufsstoffen kommt, die einzeln und/oder in Kombination hautschädigend sein können.^5^, ^13^, ^14^

Im Rahmen des Verbund‐Forschungsprojektes FP 275 wurde ein standardisiertes Testverfahren für Testungen gegen hydrophile Irritanzien (tensidisch, saurer und alkalischer Bereich) entwickelt.^15^, ^16^ Basierend auf diesen Erkenntnissen wurden „die Grundsätze zur Prüfung und Zertifizierung der Wirksamkeit von Hautschutzmittel“ der Deutschen Gesetzlichen Unfallversicherung (DGUV) formuliert.^17^, ^18^ Die Methodik überprüft die Wirksamkeit der Hautschutzmittel gegen Natriumlaurylsulfat – ein anionisches Detergens – als Modell‐Substanz für wasserlösliche hautirritierende Stoffe. Wird die Wirksamkeit eines Hautschutzmittels nachgewiesen, erhält es das DGUV‐Test Prüfzeichen GS‐PS‐14 „Wirksamkeit geprüft“, das am Produkt angebracht werden darf. Hautschutzmittel, die mit diesem Prüfzeichen ausgezeichnet sind, haben ihre Wirksamkeit gegen das Modellirritans nachgewiesen und bieten Schutz vor wässrigen Substanzen wie Seifen (typische Feuchtarbeitsplätze wie zum Beispiel im Gesundheitswesen). Eine Schutzwirkung gegen lipophile Irritanzien konnte bis dato bei keinem Präparat nachgewiesen werden.^19^

Bei bereits bestehender Sensibilisierung ist der Nutzen von Hautmitteln in der Prävention des allergischen Kontaktekzems kritisch zu betrachten.^20^

### Wirksamkeitsnachweise von Hautpflegemitteln

Hautpflegemittel sollten laut Hersteller zur „Förderung der Regeneration“ der Haut nach der Arbeit beitragen. Hierzu liegen noch keine einheitlichen beziehungsweise standardisierten Testprotokolle vor, um die barriereregenerative Wirkung der Hautpflegemittel zu überprüfen. Für die Auswahl von Hautpflegemitteln sind der Leitliniengruppe keine einheitlich standardisierten Testprotokolle bekannt, die eine barriereregenerative Wirkung der Hautpflegemittel nachweisen. In der Literatur finden sich Studien, die die Wirksamkeit von Pflegepräparaten auf verschiedene akute und kumulative Irritationen unter den Bedingungen einer täglichen Exposition untersuchten.^21^, ^22^, ^23^, ^24^, ^25^, ^26^, ^27^ Hier konnte zum Teil neben einer barriereregenerativen Wirkung, auch eine protektive Wirkung von Hautpflegepräparaten festgestellt werden.^28^, ^29^ Auch experimentelle Versuche, die eine regenerative Wirkung von Hautpflegeprodukten nachweisen konnten,^30^ wurden entwickelt, sind jedoch noch nicht validiert.

Hautpflegemittel können neben einer Glättung der Hautoberfläche und einem subjektiven Pflegegefühl auch zu einer Erhöhung des Lipid‐ und des Wassergehalts der Epidermis führen. Sie reduzieren auch die Trockenheit der Haut nach dem repetitiven Händewaschen.^31^ Hautpflegemittel sollten bei beginnenden Rötungen/Schuppungen (Zeichen eines beginnendes Handekzems) verwendet werden, da sie die gemäß der Leitlinie zur „Diagnostik, Prävention und Therapie des Handekzems“ (Registernummer 013‐053) die erforderliche Basistherapie der Handekzeme darstellen.^32^, ^33^

### Wirksamkeitsnachweise von Hautreinigungsmitteln

Eine adäquate berufliche Hautreinigung ist von Bedeutung für die Prävention beruflicher Hautkrankheiten. Die Hautreinigung ist dann als angemessen zu bezeichnen, wenn sie die Verschmutzungen, Irritanzien und Allergene effektiv und gleichzeitig hautschonend von der Hautoberfläche entfernt. Für den Anwendenden sollten nachvollziehbare Produktprüfungsmethoden und ‐resultate erhältlich sein.

Transferfähige Testverfahren für die Untersuchung der Wirksamkeit und Sicherheit beruflicher Hautreinigungsmittel wurden in Rahmen des Verbund‐Forschungsprojektes FP 276 entwickelt, indem Modellschmutze standardisiert,^34^ Modellhandreiniger entwickelt sowie ein Unterarm‐Hautwaschapparat konzipiert wurden.^35^, ^36^ Diese Methode zur Bestimmung der Reinigungswirkung wurde multizentrisch evaluiert.^37^, ^38^, ^39^ Die Testverfahren eignen sich künftig für eine standardisierte Prüfung beruflicher Hautreinigungsmittel im Vergleich zu den entwickelten Modellhandreinigern und sollen zu einer besseren Produkttransparenz beitragen.

### Empfehlungen (Wirksamkeitsnachweise)

**Table:** 

Bei der Wahl von Hautschutzmitteln zum Schutz vor wasserlöslichen Substanzen *sollten* Hautschutzmittel bevorzugt werden, deren klinische Wirksamkeit experimentell in standardisierten Testverfahren (hierzu siehe zum Beispiel Protokolle der Multicenterstudie FP275, DGUV Test Prüfzeichen) belegt wurde.	**↑**	100 % (12/12) starker Konsens
Bei der Wahl von beruflichen Hautreinigungsmitteln *sollten* möglichst solche bevorzugt werden, deren Wirksamkeit und Verträglichkeit in vivo durch kumulative Testverfahren gezeigt wurden (zum Beispiel Testverfahren aus dem Projekt FP276).	**↑**	100 % (11/11) starker Konsens *1 Enthaltung aufgrund von COI

## BEREITSTELLUNG DER HAUTSCHUTZ‐, HAUTPFLEGE‐ UND HAUTREINIGUNGSMITTEL AM ARBEITSPLATZ

### Auswahl von Hautschutz‐ und Hautpflegemitteln

Die Auswahl der Hautmittel sollte auf einer Gefährdungsbeurteilung basieren, die arbeitsplatzbezogene Expositionen und Risken berücksichtigt (siehe Kapitel 5). Die klassischen Einsatzgebiete für Hautschutzmittel sind die Feuchtarbeit sowie Tätigkeiten mit schwach hautreizenden Stoffen, wenn keine Schutzhandschuhe getragen werden dürfen oder können.^3^ Hautschutzmittel bieten keinen ausreichenden Schutz gegen oft komplex zusammengesetzte Verschmutzungen, die hautresorptive Gefahrstoffe enthalten können. Hier ist das Tragen von Schutzhandschuhen obligat.

Bei der Auswahl von Hautpflegemitteln spielt vor allem der aktuelle Hautzustand eine wesentliche Rolle. Generell werden in der Praxis Hautpflegecremes bevorzugt. Fettigere Zubereitungen können bei hoher Hauttrockenheit verwendet werden, allerdings sind Salben, als wasserfreie Grundlagen, ungeeignet und wenn überhaupt nur kurzfristig anzuwenden.^40^ Laut AWMF S2k‐Leitlinie Registernummer 013‐092 („Gebrauch von Präparationen zur lokalen Anwendung auf der Haut“) werden für eine barrieresubstituierende Basistherapie solche Präparate empfohlen, die den aktuellen Wasseranteil (Hydratation) der Haut berücksichtigen und einen Zusatz von wasserbindenden Stoffen enthalten (Urea und/oder Glycerol).^40^ Bei stärkerer Barriereschädigung sollten weitere Maßnahmen nach der S2k‐Leitlinie „Diagnostik, Prävention und Therapie des Handekzems“ eingeleitet werden.^32^

**Hautschutzmittel und flüssigkeitsdichte Handschuhe**:

Flüssigkeitsdichte Schutzhandschuhe (fSH) nach TRGS 401 („Gefährdung durch Hautkontakt, Ermittlung – Beurteilung – Maßnahmen“, Technische Regel für Gefahrstoffe, der Bundesanstalt für Arbeitsschutz und Arbeitsmedizin) sind spezialisierte Handschuhe, die einen umfassenden Schutz gegen Flüssigkeiten bieten. Sie umfassen Chemikalienschutzhandschuhe gemäß DIN EN ISO 374‐1, medizinische Handschuhe nach DIN EN 455 sowie Gewebehandschuhe mit Vollbeschichtung, beispielsweise aus Nitrilkautschuk. Gerade an Arbeitsplätzen mit Anwendung von fSH, regelmäßigen Händewaschen und zusätzlichen Kontakt zu Wasser oder irritativen Stoffen ist eine negative Beeinflussung der Hautbarriere anzunehmen. Wenn Hautschutzmittel dann zusammen mit fSH angewendet werden, sind diese im zeitlichen Abstand zu der Nutzung von Schutzhandschuhen anzuwenden, da sie unter anderem die Handschuheigenschaften beeinflussen können.^3^, ^41^ Wenn nach dem Eincremen fSH verwendet werden sollen, muss das Hautschutzmittel daher vollständig eingezogen sein. Ob die Anwendung von Hautschutzmittel unter Handschuhen einen positiven Effekt auf die Auswirkungen der Handschuhokklusion haben, kann zurzeit nicht definitiv belegt werden. Eine aktuelle experimentelle Studie weist daraufhin, dass der angenommene Okklusionseffekt durch die vor dem Tragen von fSH eingesetzten Hautschutzmittel nicht verhindert werden konnte und einige Hautschutzpräparate sogar die Hautempfindlichkeit gegenüber Tensiden zu verstärken schienen.^42^

Wenn Schutzhandschuhe nicht getragen werden dürfen, beispielsweise bei Arbeiten an rotierenden Teilen, erlaubt die TRGS 401 die Verwendung von Hautschutzmitteln zum Schutz vor schwach reizenden Stoffen. Es ist jedoch wichtig zu beachten, dass Hautschutzmittel nicht den gleichen Schutz bieten können wie fSH.

Nach Herstellerangaben könnten einige Hautschutzmittel auch die Reinigung erleichtern und somit theoretisch die Intensität und Häufigkeit aggressiver Hautreinigungsprozeduren und die Reinigungsintensität vermindern. Der Leitliniengruppe sind bisher keine wissenschaftlichen Studien bekannt, die eine Quantifizierung der Erleichterung der Reinigung durch diese Hautschutzmittel zeigen. Diese Mittel sollen aufgrund ihres hohen Emulgatoranteils nicht unter Handschuhen verwendet werden.^3^, ^43^

### Empfehlungen (Auswahl von Hautschutz‐ und Hautpflegemitteln)

**Table:** 

Die Auswahl und Anwendungsart der Hautmittel *soll* auf einer arbeitsplatz‐ und tätigkeitsbezogenen Gefährdungsbeurteilung basieren.	**↑↑**	100 % (12/12) starker Konsens
Hautschutzmittel mit erwiesener Schutzwirkung gegen die am Arbeitsplatz vorkommenden wässrigen Irritanzien *sollten* verwendet werden.	**↑**	100 % (12/12) starker Konsens
Hautschutzmittel *sollen* die Schutzhandschuhe *nicht* ersetzen.	**↓↓**	100 % (12/12) starker Konsens
Bei der Wahl der Hautmittel *sollten* der aktuelle Hautzustand, Hauttrockenheit und individuelle Prädispositionen beziehungsweise Vorerkrankungen berücksichtigt werden.	**↑**	100 % (12/12) starker Konsens

### Anwendung von Hautschutz‐ und Hautpflegemitteln

Die Hautschutz‐ und Hautpflegemittel sollten auf die trockene und saubere Haut aufgetragen werden.^3^ Die Anwendungszeitpunkte der Hautschutz‐ und Hautpflegemittel sollten in den jeweiligen Arbeitsabläufen berücksichtigt werden. Die Frage nach der ausreichenden und optimalen Anwendungsfrequenz kann nicht pauschal beantwortet werden und wurde bis dato nicht ausreichend wissenschaftlich untersucht. Sie sollte sich vielmehr an der Gefährdungsbeurteilung, der Galenik des Hautschutzmittels sowie an individuellen Faktoren orientieren. Von den Herstellern wird empfohlen, Hautschutzpräparate vor jeder Exposition mit hautirritativen Noxen sowie nach jeder Arbeitspause oder auch nach einem bestimmten Zeitraum (zum Beispiel einer halben Arbeitsschicht) aufzutragen, sofern keine tätigkeitsbezogenen Hinderungsgründe vorliegen. Dagegen können die Hautpflegepräparate in den längeren Pausen und nach der Arbeit angewendet werden.^44^

Der Einfluss einer ausreichenden Auftragsmenge an Hautschutzmittel auf die Güte der Schutzwirkung wurde bisher nur unter experimentellen Bedingungen untersucht.^39^ Unter Arbeitsbedingungen wurde an Pflegepersonal gezeigt, dass die gewählte Auftragsmenge von Hautschutzmitteln oft sehr gering ist und möglicherweise keine optimale Schutzwirkung zulässt.^45^

In der Praxis wird zur Schätzung der ausreichenden Menge des Hautschutzmittels die Fingertip‐Unit (FTU) genutzt. Dabei handelt es sich um eine Maßeinheit zur Bestimmung der Salben‐ beziehungsweise Crememenge, gemessen von der Fingerspitze bis zur distalen Interdigitalfalte. Für die Mehrheit der Hautschutz‐ und Pflegecremes (Galenik: Creme) kann nach dem generellen Konsens eine FTU pro Hand empfohlen werden.

Die Anwendung von Hautschutzmitteln zur Erleichterung der Hautreinigung führt zwar zu einer hautschonenderen Entfernung der Verschmutzung, jedoch schützen sie nicht zwangsläufig vor den Bestandteilen des Schmutzes (hierzu siehe auch Ausführungen oben). Bei Tätigkeiten, die mit einer Verschmutzung einhergehen, sind möglichst Schutzhandschuhe zu tragen.

Aus hygienischen und galenischen Gründen ist in der arbeitsmedizinischen Praxis das Verfallsdatum eines Hautmittels zu beachten (zum Beispiel bei Spendersystemen) sowie dessen Konsistenz (zum Beispiel Trennung der Phasen).

Zur Vermeidung von mikrobiologischen Kontaminationen sind Einmalspender oder Pressspendersysteme zu empfehlen. Nach der S2k‐Leitlinie Händedesinfektion und Händehygiene (Registernummer 075 – 004) sollten Hautschutz‐ und ‐pflegemittel wegen der Kontaminationsgefahr in Spendern oder Tuben bereitgestellt werden.^46^ Eine Entnahme aus Tiegeln wird aus hygienischen Gründen nicht empfohlen.

Im Zusammenhang mit einer anerkannten Berufskrankheit (BK) 5101 beziehungsweise bereits bei drohender BK (im Rahmen von § 3 Maßnahmen beziehungsweise Hautarztverfahren) können die Hautpflegepräparate als Basistherapeutika für Einzelpersonen auch von Ärzten zu Lasten des jeweiligen Unfallversicherungsträgers bei Vorliegen eines entsprechenden Behandlungsauftrags verordnet werden. Zu beachten ist, dass die ärztlich verordneten Hautmittel am Arbeitsplatz in Abstimmung mit dem Arbeitgeber verwendet werden sollten. Zudem ist eine Abstimmung mit der Sicherheitsfachkraft oder – falls möglich – dem Betriebsarzt empfohlen.

### Empfehlungen (Anwendung von Hautschutz‐ und Hautpflegemitteln)

**Table:** 

Die Hautschutz‐ und Hautpflegemittel *sollen* in ausreichender Menge und Frequenz aufgetragen werden. Dabei *sollen* die produktspezifischen Empfehlungen berücksichtigt werden.	**↑↑**	100 % (13/13) starker Konsens
Für die Mehrheit der Hautschutz‐ und Pflegecremes *sollte* die Menge für die Oberfläche einer Hand mindestens eine Fingertip‐Unit (FTU) betragen.	**↑**	100 % (12/12) starker Konsens
Ein Hautschutzmittel *sollte* – falls laut Gefährdungsbeurteilung indiziert – vor der hautbelastenden Tätigkeit aufgetragen werden.	**↑**	100 % (13/13) starker Konsens
Hautpflegemittel *sollen* nach der Arbeit angewendet werden.	**↑↑**	100 % (13/13) starker Konsens
Hautschutzmittel *sollten* möglichst *nicht* unmittelbar vor dem Anziehen von flüssigkeitsdichten Schutzhandschuhen verwendet werden, da sie u.a. die Handschuheigenschaften beeinflussen können.	**↓**	100 % (13/13) starker Konsens

### Auswahl und Anwendung von Hautreinigungsmitteln

Auswahl und Zusammensetzung der Hautreinigungsmittel hängen grundsätzlich von der Art der Verschmutzung (einfach, grob, spezial) ab, wobei ein entsprechender Stufenplan in Abhängigkeit vom Verschmutzungsgrad beachtet werden muss. Die Intensität der Reinigung und die Auswahl des Reinigungsmittels sind dem Grad der Verschmutzung anzupassen.^3^ Beim Einsatz von Hautreinigungsmitteln ist zu beachten, dass auch bei Produkten mit vergleichbarer Reinigungswirkung gravierende individuelle Unterschiede in der Hautverträglichkeit bestehen können.^47^

Händereinigung führt nachweislich zur Schädigung der Hautbarriere und bei zu hoher Handwaschfrequenz zu irritativen Kontaktekzemen.^48^, ^49^, ^50^ Aus präventiver Sicht sollte auf die Waschfrequenz,^51^ die Waschdauer, die Zusammensetzung der Reinigungsmittel,^52^ sowie die Verwendung der Hilfsmittel (zum Beispiel Handbürsten) geachtet werden.^53^ Die Häufigkeit des Händewaschens sollte auf ein Minimum reduziert werden, soweit dies den Hygienevorschriften entspricht. Händewaschen kommt vor allem dann in Betracht, wenn sichtbare Verschmutzungen vorliegen. Nach bisherigem Kenntnisstand ist die irritative Potenz aus chemischer Sicht vor allen Dingen vom eingesetzten Detergenstyp (anionisch, kationisch, amphoter, nicht‐ionisch) abhängig.^54^, ^55^, ^56^ Welche Rolle im Verhältnis dazu der pH‐Wert für die Hautverträglichkeit von Hautreinigungsmitteln spielt, ist nicht sicher bekannt. Aus früheren Studien geht hervor, dass die Reiniger eher einen neutralen oder sauren pH‐Wert, als einen alkalischen pH‐Wert aufweisen sollten.^54^, ^57^, ^58^, ^59^, ^60^, ^61^

In der Praxis sollten milde Reinigungsmittel, die keine Reibekörper enthalten, bevorzugt werden.^3^

Sollte eine starke Verschmutzung vorliegen, die durch den Einsatz von milden Reinigungsmitteln nicht beseitigt werden kann, können reibekörperhaltige Mittel zum Einsatz kommen. Die Reinigung der Hände mit reibekörperhaltigen Mitteln sollte auf ein Minimum reduziert werden, zum Beispiel wenn möglich am Ende des Arbeitstages. Auf den Einsatz von Bürsten sollte wenn möglich verzichtet werden. Starke Verschmutzungen können auch bereits mit Papiertüchern reduziert werden, wodurch die Anwendung der Bürsten vermieden werden kann. Hier sollten dann moderne Reibekörper aus biologischen Materialien bevorzugt werden.^62^, ^63^ Die Anwendung von harten und abrasiven Bürsten wird nicht empfohlen.^53^

Die Anwendung von antiseptischen Seifen kann generell nicht empfohlen werden. Sie können die Händedesinfektion durch alkoholische Desinfektionsmittel nicht ersetzen.^64^

### Empfehlungen (Auswahl und Anwendung von Hautreinigungsmitteln)

**Table:** 

Die Auswahl des Hautreinigungsmittels *soll* an den Verschmutzungsgrad und den Hautzustand angepasst werden.	**↑↑**	91,7 % (12/13) Konsens
Die Handwaschfrequenz *soll* auf das notwendige Minimum reduziert werden.	**↑↑**	100 % (13/13) starker Konsens

## SICHERHEITSBEWERTUNG UND UNERWÜNSCHTE WIRKUNGEN

### Sicherheitsbewertung in der Kosmetologie

Berufliche Hautmittel im Sinne dieser Leitlinie sind in Deutschland rechtlich als Kosmetika reguliert. Kosmetika sind nach der Verordnung (EG) Nr.1223/2009 des Europäischen Parlaments und des Rates vom 30.November 2009 („EU‐Kosmetik‐Verordnung“) definiert als „Stoffe oder Gemische, die dazu bestimmt sind, äußerlich mit den Teilen des menschlichen Körpers (Haut, Behaarungssystem, Nägel, Lippen und äußere intime Regionen) oder mit den Zähnen und den Schleimhäuten der Mundhöhle in Berührung zu kommen, und zwar zu dem ausschließlichen oder überwiegenden Zweck, diese zu reinigen, zu parfümieren, ihr Aussehen zu verändern, sie zu schützen, sie in gutem Zustand zu halten oder den Körpergeruch zu beeinflussen“.^65^ Die Verordnung gilt unmittelbar in allen Mitgliedstaaten der Europäischen Union und so auch in Deutschland.

Nach der EU‐Kosmetik‐Verordnung müssen „kosmetische Mittel bei normaler oder vernünftigerweise vorhersehbarer Verwendung für die menschliche Gesundheit sicher sein“ Vorgaben zur Sicherheitsbewertung von kosmetischen Inhaltsstoffen hat das *Scientific Committee on Consumer Safety* (SCCS), das im Auftrag der EU‐Kommission tätig ist, vorgelegt; diese werden regelmäßig aktualisiert.^66^

### Sensibilisierungen durch Hautmittel

Die Kosmetik‐Verordnung (EG Nr.1223/2009 2023), welcher Hautschutzmittel als kosmetische Mittel unterliegen, wird in Übereinstimmung mit entsprechenden EU‐Verordnungen ständig dem aktuellen Stand der Wissenschaft angepasst. So wurde zum Beispiel vor kurzem mit EU‐Verordnung 2023/1545 die Liste der deklarationspflichtigen Duftstoffel auf 81 Kennzeichnungspflichtige erweitert.

Da Hautmittel auch auf vorgeschädigter Haut angewendet werden, ist theoretisch das Risiko für eine Sensibilisierung gegen einzelne Inhaltsstoffe erhöht. In der wissenschaftlichen Literatur gibt es jedoch keine aktuellen entsprechenden Untersuchungen an größeren Patientengruppen, sondern lediglich Einzelfallberichte. Studien zu Sensibilisierungen durch Hautmittel sind dadurch erschwert, dass die Inhaltsstoffe von beruflich verwendeten Hautmitteln sich nicht grundsätzlich von denen privat verwendeter kosmetischer Mittel unterscheiden, sodass meist nicht zu klären ist, wodurch eine festgestellte Sensibilisierung erworben wurde.

In der Leitliniengruppe wurden Argumente für und gegen die Nutzung duftstoffhaltiger Hautmittel in Bezug auf Sensibilisierung und Irritation diskutiert. Basierend auf der aktuellen Studienlage und Erfahrungen im klinischen Alltag, ist durch die Leitliniengruppe keine Empfehlung zu dieser komplexen Thematik zu formulieren, da kein Konsens hergestellt werden konnte. Um eine Empfehlung formulieren zu können, ist weiterführende Forschung zur Nutzung von Duftstoffen in Hautmitteln notwendig.

Tabelle 2 zeigt Substanzen, die in Studien häufiger mit Kontaktallergien in Verbindung gebracht werden und in Kosmetika verwendet werden können. Der Fokus liegt dabei auf Konservierungsmitteln sowie Duftstoffen und natürlichen Extrakten. Als primäres Kriterium wurde die Reaktionsquote von über 1 % herangezogen (auch bei gezielten Epikutantestungen). Die Tabelle 2 erhebt keinen Anspruch auf Vollständigkeit. Falls die Bezeichnungen nicht identisch sind, werden die Namen gemäß der *International Nomenclature of Cosmetic Ingredients* (INCI) in Klammern angegeben.

**TABELLE 2:** In Hautmitteln aufgrund des erhöhten Risikos einer Kontaktsensibilisierung, insbesondere bei vorgeschädigter Haut, nicht empfohlene Inhaltsstoffe.

Name (INCI *)	
***Konservierungsmittel***	100 % (12/12) starker Konsens 1 Enthaltung aufgrund von Interessenkonflikten
Chlormethylisothiazolinon (Chloromethylisothiazolinone)	
Methylisothiazolinon (Methylisothiazolinone)	
***Duftstoffe***	
Citral	
Eugenol	
Farnesol	
Geraniol	
Hydroxycitronellal	
Isoeugenol	
Zimtaldehyd Cinnamal	
Zimtalkohol (Cinnamyl Alcohol)	
***Naturstoff‐Extrakte/Ätherische Öle***	
Jasmin absolut (Jasminum Officinale [Jasmine] Extract)	
Lemongrasöl (Cymbopogon Schoenanthus Oil)	
Nelkenöl (Eugenia Caryophyllus [Clove] Leaf Oil)	
Perubalsam/Myroxylon pereirae (Balsam Peru)	
Propolis (Propolis Extract)	
Sandelholzöl (Santalum Album [Sandalwood] Oil)	
Ylang‐Ylang Öl (Cananga Odorata Flower Oil)	

### Empfehlungen (Sensibilisierungen und Irritationen durch Hautmittel)

**Table:** 

Inhaltsstoffe der Tabelle 2 *sollten* speziell bei vorgeschädigter Haut in beruflichen Hautmitteln *nicht* enthalten sein.	**↓**	100 % (11/11) starker Konsens
Bei der Auswahl des Hautmittels *sollen* individuelle Sensibilisierungen berücksichtigt werden.	**↑↑**	100 % (12/12) starker Konsens

### Mögliche irritative Effekte der beruflichen Hautmittel

*Für Kapitel 5.4 „Mögliche irritative Effekte der beruflichen Hautmittel“ siehe Langfassung*.

### Händereinigung versus Händedesinfektion bei infektionsgefährdender Tätigkeit

Händedesinfektionsmittel sollte laut Herstellerangaben in ausreichender Menge (meistens 3–5 mL) auf den Händen verteilt und entsprechend lange einwirken gelassen werden. Überschüssiges Händedesinfektionsmittel ist dabei nicht abzuwischen, sondern soll verdunsten.^46^

Handhygienemaßnahmen zielen darauf ab, die Kontamination der Haut mit biologischen Krankheitserregern (Viren, Bakterien oder Parasiten) durch deren mechanische oder chemische Ablösung zu reduzieren; eine völlige Sterilität der Haut lässt sich aufgrund des physiologischerweise vorhandenen Mikrobioms nicht erreichen.^67^

Von verschiedenen dermatologischen Forschungsgruppen^68^, ^69^, ^70^, ^71^ wurden in den vergangenen Jahren Studien zur irritativen Potenz beziehungsweise zur Hautverträglichkeit von Maßnahmen zur Handhygiene durchgeführt. Dabei zeigte sich unter anderem, dass Detergenzieneffekte sich durch nachfolgende Handschuhokklusion verstärken, dies aber für alkoholische Desinfizientien nicht der Fall ist.^71^ Die kumulative irritative Potenz der alkoholischen Desinfizientien ist gering und wesentlich geringer als die von Detergenzien.^2^, ^72^, ^73^ Unter dem Aspekt der Erhaltung der Hautgesundheit ist bei nicht sichtbarer Verschmutzung, die Händedesinfektion dem Händewaschen vorzuziehen.^3^, ^68^, ^74^ Für eine Verminderung der alkoholischen Antisepsis durch Hautpflege gibt es keine Evidenz.^75^

### Empfehlungen (Händereinigung versus Händedesinfektion bei infektionsgefährdender Tätigkeit)

**Table:** 

Bei infektionsgefährdenden Tätigkeiten *sollte*, soweit möglich, bei nicht sichtbarer Verschmutzung der Haut eine Desinfektion mit Händedesinfektionsmittel der Hautreinigung mit Detergenzien vorgezogen werden.	**↑**	100 % (11/11) starker Konsens

### Sicherheitsbewertung für Aluminiumchlorohydrat in beruflichen Hautmitteln

Das Bundesinstitut für Risikobewertung (BfR) hat seine Sicherheitsbewertung für Aluminiumchlorohydrat von 2020 revidiert.^76^ Die Aluminiumaufnahme aus Antitranspirantien sei unter Beachtung einer neuen Studie zur Aufnahme von Aluminium über die Haut wesentlich niedriger als bisher angenommen. Der Beitrag dieser Aufnahmequelle zur Aluminiumgesamtaufnahme ist folglich sehr gering.

Der wissenschaftliche Ausschuss für Verbrauchersicherheit der Europäischen Kommission (SCCS) hat seine Bewertung umfangreicher neuer Daten zur Aufnahme von Aluminiumchlorohydrat aus Antitranspirantien durch die Haut veröffentlicht. Unter Heranziehung der aktuellen Humandaten zur Hautabsorption von Aluminium unter realistischen Anwendungsbedingungen,^77^ kommt die SCCS zu dem Schluss, dass Verbraucherinnen und Verbraucher signifikant weniger Aluminium aufnehmen als auf Basis der ursprünglich zur Verfügung stehenden Daten angenommen.^66^

### Empfehlungen (Sicherheitsbewertung für Aluminiumchlorohydrat in beruflichen Hautmitteln)

**Table:** 

Die Anwendung eines Hautschutz‐ oder Hautpflegemittels mit Aluminiumchlorohydrat *kann* bei intakter Haut erwogen werden.	**<–>**	81,8 % (9/11) Konsens

### Beeinflussung der Penetration von Fremdstoffen durch Hautschutzmittel

Das intakte Stratum corneum bildet die eigentliche Barriere gegen eine dermale Penetration von chemischen Stoffen. Erst wenn das Stratum corneum durchdrungen wurde, kann ein Stoff resorbiert werden.^78^ Ansonsten können vor allem kleine Moleküle auch über die Haarfollikel in die Haut gelangen.^79^

Die Penetration eines Stoffes durch die Haut kann durch die Anwesenheit weiterer Stoffe begünstigt werden. Für die Penetrationsförderung von Gefahrstoffen durch Hautschutzmittel werden Zusatzstoffe wie Emulgatoren verantwortlich gemacht.^80^, ^81^, ^82^, ^83^

Hautschutzmittel sollten nur zum Schutz der Haut vor Irritation und nicht zum Schutz vor der perkutanen Aufnahme systemtoxischer Arbeitsstoffe verwendet werden.

Experimentelle Studien zeigten, dass die Penetration von hydrophilen und lipophilen Lösungsmitteln, durch die mit Hautschutzcremes vorbehandelte Humanhaut nicht reduziert,^84^, ^85^ sondern in der Regel gefördert wird.^81^, ^82^, ^83^, ^84^, ^86^, ^87^

Zusammenfassend kann von einer generellen Reduzierung der perkutanen Aufnahme von Fremdstoffen durch die Applikation von Hautschutzmitteln vor der Exposition nicht ausgegangen werden.

### Empfehlungen (Beeinflussung der Penetration von Fremdstoffen durch Hautschutzmittel)

**Table:** 

Die Gefahr einer möglichen Penetrationsbeschleunigung der Gefahrstoffe/Fremdstoffe durch Hautschutzmittel *soll* bei deren Einsatz und Auswahl berücksichtigt werden.	**↑↑**	100 % (12/12) starker Konsens
Hautschutzmittel *sollen nicht* zum Schutz vor der perkutanen Aufnahme systemtoxischer Arbeitsstoffe, zum Beispiel krebserzeugender oder hautresorptiver Stoffe verwendet werden.	**↓↓**	100 % (11/11) starker Konsens

### Direkter Kontakt zu patienteneigenen Pflegemitteln und kritische Nutzung der Schutzhandschuhe im Gesundheitswesen. Kritischer Einsatz von Einmalhandschuhen im Gesundheitswesen

Die Kommission für Krankenhaushygiene und Infektionsprävention (KRINKO) betont in einer veröffentlichten KRINKO‐Empfehlung die Notwendigkeit eines ausgewogenen Einsatzes von Einmalhandschuhen, um sowohl den Hygieneschutz zu gewährleisten als auch ökologische und gesundheitliche Aspekte zu berücksichtigen.^88^ Allerdings beinhaltet die KRINKO‐Empfehlung zwei Tätigkeiten, die aus Sicht der Autoren unter Schutz durch flüssigkeitsdichte Handschuhe erfolgen sollten, nämlich *(1)* Körperwaschungen und *(2)* das Eincremen von Patienten.

**Table:** 

Im Gesundheitswesen *sollten* flüssigkeitsdichte Handschuhe bei Körperwaschungen getragen werden, um den Hautkontakt mit Reinigungsmitteln und Wasser zu reduzieren.	**↑**	100 % (11/11) starker Konsens
Beim Auftragen von Pflegeprodukten auf die Haut von Patienten *sollten* flüssigkeitsdichte Handschuhe getragen werden, um einen direkten Kontakt mit möglicherweise sensibilisierenden Inhaltsstoffen und die Handwaschfrequenz zu reduzieren.	**↑**	100 % (11/11) starker Konsens

*Für die Abschnitte „Dokumentationsempfehlungen an Hersteller“, „Ausblicke und Forschungsbedarf“, „Limitationen der Leitlinie“, „Informationen zu dieser Leitlinie“ (inklusive „Umgang mit Interessenskonflikten“) sowie „Methodik“ (inklusive „Generierung von Empfehlungen/Konsensuskonferenz“, „Verabschiedung der Leitlinie“ und „Verwertungsrechte“) siehe Langfassung*.

## DANKSAGUNG

Open access Veröffentlichung ermöglicht und organisiert durch Projekt DEAL.

## INTERESSENKONFLIKT

Eine vollständige Auflistung der angegebenen Interessenkonflikte ist im Leitliniendokument verfügbar unter https://register.awmf.org/de/leitlinien/detail/013‐056.

## References

[1] Fartasch M , Diepgen T , Drexler H , et al. Berufliche Hautmittel: S1‐Leitlinie der Arbeitsgemeinschaft für Berufs‐ und Umweltdermatologie (ABD) in der Deutschen Dermatologischen Gesellschaft (DDG). Arbeitsmed Sozialmed Umweltmed. 2009;44:53‐67.

[2] Fartasch M , Diepgen TL , Drexler H , et al. S1 guideline on occupational skin products: protective creams, skin cleansers, skin care products (ICD 10: L23, L24) – short version. J Dtsch Dermatol Ges. 2015;13:594‐606. 25997664 10.1111/ddg.12617

[3] Bundesanstalt für Arbeitsschutz und Arbeitsmedizin . TRGS 401 Gefährdung durch Hautkontakt, Ermittlung – Beurteilung – Maßnahmen. 06 September 2024. Available from: https://www.baua.de/DE/Angebote/Regelwerk/TRGS/TRGS‐401 [Last accessed June 26, 2024].

[4] Kaminski‐Hartenthaler A , Meerpohl JJ , Gartlehner G , et al. [GRADE guidelines: 14. Going from evidence to recommendations: the significance and presentation of recommendations]. Z Evid Fortbild Qual Gesundhwes. 2014;108:413‐420. 25444300 10.1016/j.zefq.2014.08.003

[5] Wigger‐Alberti W . Möglichkeiten und Grenzen von Hautschutzmitteln. Dermatol Beruf Umwelt. 2005;53:158‐166.

[6] Wigger‐Alberti W , Diepgen T , Elsner P , et al. Berufliche Hautschutzmittel. Gemeinsame Leitlinie der Arbeitsgemeinschaft für Berufs‐ und Umweltdermatologie (ABD) in der Deutschen Dermatologischen Gesellschaft (DDG) und der Gesellschaft für Dermopharmazie e.V. (GD). Dermatol Beruf Umwelt. 2003;51:15‐21.

[7] Kresken J , Klotz A . Occupational skin‐protection products – a review. Int Arch Occup Environ Health. 2003;76:355‐358. 12856190 10.1007/s00420-002-0422-5

[8] Schliemann‐Willers S , Elsner P . [Occupational skin protection] Beruflicher Hautschutz. J Dtsch Dermatol Ges. 2005;3:120‐133; quiz 134‐136. 16351016 10.1111/j.1610-0378.2005.04531.x

[9] Wigger‐Alberti W , Elsner P . Preventive measures in contact dermatitis. Clin Dermatol. 1997;15:661‐665. 9255478 10.1016/s0738-081x(97)00001-1

[10] Alfonso JH , Bauer A , Bensefa‐Colas L , et al. Minimum standards on prevention, diagnosis and treatment of occupational and work‐related skin diseases in Europe ‐ position paper of the COST Action StanDerm (TD 1206). J Eur Acad Dermatol Venereol. 2017;31(Suppl 4):31‐43. 28656728 10.1111/jdv.14319

[11] Bauer A , Rönsch H , Elsner P , et al. Interventions for preventing occupational irritant hand dermatitis. Cochrane Database Syst Rev. 2018;4:Cd004414. 29708265 10.1002/14651858.CD004414.pub3 PMC6494486

[12] Coenraads PJ , Diepgen TL . Problems with trials and intervention studies on barrier creams and emollients at the workplace. Int Arch Occup Environ Health. 2003;76:362‐366. 12768427 10.1007/s00420-002-0424-3

[13] Fluhr JW , Akengin A , Bornkessel A , et al. Additive impairment of the barrier function by mechanical irritation, occlusion and sodium lauryl sulphate in vivo. Br J Dermatol. 2005;153:125‐131. 16029337 10.1111/j.1365-2133.2005.06430.x

[14] Kappes UP , Göritz N , Wigger‐Alberti W , et al. Tandem application of sodium lauryl sulfate and n‐propanol does not lead to enhancement of cumulative skin irritation. Acta Derm Venereol. 2001;81:403‐405. 11859941 10.1080/000155501317208327

[15] Schliemann S , Müller M , Stadeler M , Elsner P . Doppelblinde, randomisierte repetitive In‐vivo‐Wirksamkeitsstudie zu kommerziellen Hautschutzprodukten gegen Natriumlaurylsulfat (SLS). J Dtsch Dermatol Ges. 2021;19:545‐553. 10.1111/ddg.14359_g 33861011

[16] Schliemann S , Elsner P , Scheidt R . In‐vivo‐Evaluationsmodelle zur Überprüfung der Wirkung von Hautschutzexterna: Bestimmung der schützenden Wirksamkeit und Vergleichbarkeit, FP275. Abschlußbericht. Unter Mitarbeit von Peter Elsner, Hans Drexler, T. L. Diepgen, Sibylle Schliemann, S. M. John, Reginald Scheidt etal. Hg. v. DGUV‐Deutsche Gesetzliche Unfallversicherung. 24 September 2021; Available from: https://www.dguv.de/projektdatenbank/0275/abschlussberichtfp275final03.pdf [Last accessed June 26, 2024].

[17] DGUV . Hautschutzmittel, die sicher schützen. 19 April 2023; Available from: https://www.dguv.de/dguv‐test/aktuelles/2021/2021_details_448768.jsp [Last accessed June 26, 2024].

[18] Pieper B . Grundsätze zur Prüfung und Zertifizierung der Wirksamkeit von Hautschutzmitteln. sicher ist sicher. 2021;72:318‐319.

[19] Schliemann S , Kleesz P , Elsner P . Protective creams fail to prevent solvent‐induced cumulative skin irritation – results of a randomized double‐blind study. Contact Dermatitis. 2013;69:363‐371. 23844768 10.1111/cod.12103

[20] Schliemann S , Wigger‐Alberti W , Elsner P . Prevention of allergy by protective skin creams: possibilities and limits. Schweiz Med Wochenschr. 1999;129:996‐1001. 10431324

[21] El Gammal C , Pagnoni A , Kligman AM , el Gammal S . A model to assess the efficacy of moisturizers–the quantification of soap‐induced xerosis by image analysis of adhesive‐coated discs (D‐Squames). Clin Exp Dermatol. 1996;21:338‐343. 9136151

[22] Halkier‐Sørensen L , Thestrup‐Pedersen K . The efficacy of a moisturizer (Locobase) among cleaners and kitchen assistants during everyday exposure to water and detergents. Contact Dermatitis. 1993;29:266‐271. 8112068 10.1111/j.1600-0536.1993.tb03563.x

[23] Hannuksela A , Kinnunen T . Moisturizers prevent irritant dermatitis. Acta Derm Venereol. 1992;72:42‐44. 1350143

[24] Lodén M . Barrier recovery and influence of irritant stimuli in skin treated with a moisturizing cream. Contact Dermatitis. 1997;36:256‐260. 9197961 10.1111/j.1600-0536.1997.tb00213.x

[25] Lodén M , Andersson AC . Effect of topically applied lipids on surfactant‐irritated skin. Br J Dermatol. 1996;134:215‐220. 8746332

[26] Mortz CG , Andersen KE , Halkier‐Sørensen L . The efficacy of different moisturizers on barrier recovery in hairless mice evaluated by non‐invasive bioengineering methods. A model to select the potentially most effective product. Contact Dermatitis. 1997;36:297‐301. 9237008 10.1111/j.1600-0536.1997.tb00004.x

[27] Treffel P , Gabard B . Stratum corneum dynamic function measurements after moisturizer or irritant application. Arch Dermatol Res. 1995;287:474‐479. 7625859 10.1007/BF00373431

[28] Buraczewska I , Berne B , Lindberg M , et al. Changes in skin barrier function following long‐term treatment with moisturizers, a randomized controlled trial. Br J Dermatol. 2007;156:492‐498. 17300239 10.1111/j.1365-2133.2006.07685.x

[29] Ramsing DW , Agner T . Preventive and therapeutic effects of a moisturizer. An experimental study of human skin. Acta Derm Venereol. 1997;77:335‐337. 9298122 10.2340/0001555577335337

[30] Elsner P , Babin M , Sohm B , et al. Wirksamkeit eines topischen Medizinprodukts in der Behandlung des kumulativen irritativen Kontaktekzems. Dermatol. Beruf Umwelt. 2020;68:131‐140.

[31] Kampf G , Ennen J . Regular use of a hand cream can attenuate skin dryness and roughness caused by frequent hand washing. BMC Dermatol. 2006;6:1. 16476166 10.1186/1471-5945-6-1 PMC1397860

[32] Bauer A , Brans R , Brehler R , et al. [S2k‐Leitlinie Diagnostik, Prävention und Therapie des Handekzems: S2k guideline diagnosis, prevention and therapy of hand eczema]. J Dtsch Dermatol Ges. 2023;21:1054‐1076. 10.1111/ddg.15179_g 37700403

[33] van Zuuren EJ , Fedorowicz Z , Christensen R , et al. Emollients and moisturisers for eczema. Cochrane Database Syst Rev. 2017;2:Cd012119. 28166390 10.1002/14651858.CD012119.pub2 PMC6464068

[34] Elsner P , Seyfarth F , Sonsmann F , et al. Standardized dirts for testing the efficacy of workplace cleaning products: validation of their workplace relevance. Contact Dermatitis. 2013;69:245‐250. 23808852 10.1111/cod.12094

[35] Sonsmann FK , Strunk M , Gediga K , et al. Standardization of skin cleansing in vivo: part I. Development of an Automated Cleansing Device (ACiD). Skin Res Technol. 2014;20:228‐238. 24138130 10.1111/srt.12112

[36] Sonsmann FK , Strunk M , Gediga K , et al. Standardization of skin cleansing in vivo: part II. Validation of a newly developed Automated Cleansing Device (ACiD). Skin Res Technol. 2014;20:239‐245. 24003846 10.1111/srt.12113

[37] Elsner P , Seyfarth F , Antonov D , et al. Development of a standardized testing procedure for assessing the irritation potential of occupational skin cleansers. Contact Dermatitis. 2014;70:151‐157. 24588368 10.1111/cod.12140

[38] Elsner P , Seyfarth F , Sonsmann F , et al. Development of a standardized procedure for testing the efficacy of workplace cleansers. Contact Dermatitis. 2014;70:35‐43. 24102685 10.1111/cod.12121

[39] Schliemann S , Diepgen TL , John SM , et al. In‐vivo‐Evaluierung von Hautreinigungsprodukten – Wissenschaftlicher Abschlussbericht des DGUV‐Forschungsprojektes FP 276. Derm Beruf Umwelt. 2014; 62:3‐34.

[40] Wohlrab J , Staubach P , Augustin M , et al. S2k‐Leitlinie zum Gebrauch von Präparationen zur lokalen Anwendung auf der Haut (Topika). J Dtsch Dermatol Ges. 2018;16:376‐392. 10.1111/ddg.13473_g 29537157

[41] Schliemann S . Adverse Effects of Skin Protective Products, Including Sunscreens. In: Maibach F , ed. Kanerva's Occupational Dermatology. Springer International Publishing; 2018:1‐6.

[42] Gina M , Wichert K , Kutz G , et al. Applying skin protective cream and the wearing of gloves?‐A randomized controlled experimental study. Contact Dermatitis. 2023;88:372‐382. 36727715 10.1111/cod.14287

[43] DGUV . DGUV Information 212‐017: Auswahl, Bereitstellung und Benutzung von beruflichen Hautmitteln. 07/03/2025; Available from: https://publikationen.dguv.de/regelwerk/dguv‐informationen/853/auswahl‐bereitstellung‐und‐benutzung‐von‐beruflichen‐hautmitteln [Last accessed June 26, 2024].

[44] Hines J , Wilkinson SM , John SM , et al. The three moments of skin cream application: an evidence‐based proposal for use of skin creams in the prevention of irritant contact dermatitis in the workplace. J Eur Acad Dermatol Venereol. 2017;31:53‐64. 27545662 10.1111/jdv.13851 PMC5434821

[45] Schliemann S , Petri M , Elsner P . How much skin protection cream is actually applied in the workplace? Determination of dose per skin surface area in nurses. Contact Dermatitis. 2012;67:229‐233. 22709142 10.1111/j.1600-0536.2012.02119.x

[46] Kramer A , Seifert J , Abele‐Horn M , et al. S2k‐Leitlinie "Händedesinfektion und Händehygiene". Krankenhaushygiene, Registernummer 075 ‐ 004. Gesellschaft für Krankenhaushygiene. 12 September 2023; Available from: https://register.awmf.org/de/leitlinien/detail/075‐004 [Last accessed June 26, 2024].

[47] Klotz A , Veeger M , Röcher W . Skin cleansers for occupational use: testing the skin compatibility of different formulations. Int Arch Occup Environ Health. 2003;76:367‐373. 12768428 10.1007/s00420-002-0427-0

[48] Berardesca E , Vignoli GP , Distante F , et al. Effects of water temperature on surfactant‐induced skin irritation. Contact Dermatitis. 1995;32:83‐87. 7758326 10.1111/j.1600-0536.1995.tb00751.x

[49] Carøe TK , Ebbehøj NE , Bonde JPE , et al. Hand eczema and wet work: dose‐response relationship and effect of leaving the profession. Contact Dermatitis. 2018;78:341‐347. 29508401 10.1111/cod.12934

[50] Hamnerius N , Svedman C , Bergendorff O , et al. Wet work exposure and hand eczema among healthcare workers: a cross‐sectional study. Br J Dermatol. 2018;178:452‐461. 28722122 10.1111/bjd.15813

[51] Symanzik C , Kezic S , Jakasa I , et al. Effects of skin washing frequency on the epidermal barrier function and inflammatory processes of the epidermis: An experimental study. Contact Dermatitis. 2022;87:241‐246. 35357722 10.1111/cod.14119

[52] Larson E , Girard R , Pessoa‐Silva CL , et al. Skin reactions related to hand hygiene and selection of hand hygiene products. Am J Infect Control. 2006;34:627‐635. 17161737 10.1016/j.ajic.2006.05.289

[53] Gina M , Wichert K , Pieper B , et al. Irritant potential of different washing procedures used for heavy‐duty soiling: Short and intense or longer and mild? Contact Dermatitis. 2023;88:363‐371. 36727255 10.1111/cod.14282

[54] Ananthapadmanabhan KP , Moore DJ , Subramanyan K , et al. Cleansing without compromise: the impact of cleansers on the skin barrier and the technology of mild cleansing. Dermatol Ther. 2004;17(Suppl 1):16‐25. 14728695 10.1111/j.1396-0296.2004.04s1002.x

[55] Corazza M , Lauriola MM , Bianchi A , et al. Irritant and sensitizing potential of eight surfactants commonly used in skin cleansers: an evaluation of 105 patients. Dermatitis. 2010;21:262‐268. 20920412

[56] Corazza M , Lauriola MM , Zappaterra M , et al. Surfactants, skin cleansing protagonists. J Eur Acad Dermatol Venereol. 2010;24:1‐6. 10.1111/j.1468-3083.2009.03349.x 19614860

[57] Baranda L , González‐Amaro R , Torres‐Alvarez B , et al. Correlation between pH and irritant effect of cleansers marketed for dry skin. Int J Dermatol. 2002;41:494‐499. 12207765 10.1046/j.1365-4362.2002.01555.x

[58] Korting HC , Hübner K , Greiner K , et al. Differences in the skin surface pH and bacterial microflora due to the long‐term application of synthetic detergent preparations of pH 5.5 and pH 7.0. Results of a crossover trial in healthy volunteers. Acta Derm Venereol. 1990;70:429‐431. 1980979

[59] Korting HC , Ponce‐Pöschl E , Klövekorn W , et al. The influence of the regular use of a soap or an acidic syndet bar on pre‐acne. Infection. 1995;23:89‐93. 7622270 10.1007/BF01833872

[60] Park KS , Kim YS , Cho YH , et al. Effects of alkalinity of household dishwashing liquids on hand skin. Contact Dermatitis. 2001;45:95‐98. 11553119 10.1034/j.1600-0536.2001.045002095.x

[61] Schmid‐Wendtner M , Korting HC . pH and skin care. ABW Wissenschaftsverlag; 2007.

[62] Mahler V , Erfurt‐Berge C , Schiemann S , et al. Dirt‐binding particles consisting of hydrogenated castor oil beads constitute a nonirritating alternative for abrasive cleaning of recalcitrant oily skin contamination in a three‐step programme of occupational skin protection. Br J Dermatol. 2010;162:812‐818. 19995365 10.1111/j.1365-2133.2009.09602.x

[63] Terhaer FK , Bock M , Fartasch M , et al. Safety, effectiveness and comparability of professional skin cleansers. J Dtsch Dermatol Ges. 2010;8:806‐810. 20561116 10.1111/j.1610-0387.2010.07468.x

[64] Kampf G . Bedeutung und Häufigkeit von Toleranzen gegenüber bioziden Wirkstoffen in Antiseptika und Desinfektionsmitteln. Epidemiologisches Bulletin. 2020;39:3‐11.

[65] Europäisches Parlament . Verordnung (EG) Nr. 1223/2009 des Europäischen Parlaments und des Rates vom 30. November 2009 über kosmetische Mittel (Neufassung).

[66] Scientific Committee on Consumer Safety (SCCS) . SCCS Notes of guidance for the testing of cosmetic ingredients and their safety evaluation –12th revision. 24 May 2023; Available from: https://health.ec.europa.eu/publications/sccs‐notes‐guidance‐testing‐cosmetic‐ingredients‐and‐their‐safety‐evaluation‐12th‐revision_en [Last accessed June 26, 2024].

[67] Pothmann A , Illing T , Wiegand C , et al. The Microbiome and Atopic Dermatitis: A Review. Am J Clin Dermatol. 2019;20:749‐761. 31444782 10.1007/s40257-019-00467-1

[68] Pedersen LK , Held E , Johansen JD , Agner T . Less skin irritation from alcohol‐based disinfectant than from detergent used for hand disinfection. Br J Dermatol. 2005;153:1142‐1146. 16307649 10.1111/j.1365-2133.2005.06875.x

[69] Slotosch CM , Kampf G , Löffler H . Effects of disinfectants and detergents on skin irritation. Contact Dermatitis. 2007;57:235‐241. 17868216 10.1111/j.1600-0536.2007.01200.x

[70] Wigger‐Alberti W , Spoo J , Schliemann‐Willers S , et al. The tandem repeated irritation test: a new method to assess prevention of irritant combination damage to the skin. Acta Derm Venereol. 2002;82:94‐97. 12125960 10.1080/00015550252948103

[71] Welle S . Vergleichende Untersuchungen zum kumulativen in‐vivo Irritationspotential beruflich relevanter alkoholischer Irritanzien . Friedrich‐Schiller‐Universität Jena; 2019.

[72] Diepgen TL , Andersen KE , Chosidow O , et al. Guidelines for diagnosis, prevention and treatment of hand eczema. J Dtsch Dermatol Ges. 2015;13:e1‐e22. 25763418 10.1111/ddg.12510_1

[73] Elsner P , Fartasch M , Schliemann S . Dermatologische Empfehlungen zur Handhygiene in Schulen während der COVID‐19‐Pandemie. J Dtsch Dermatol Ges. 2020;18:892‐894. 10.1111/ddg.14170_g PMC7461413 32881261

[74] Jungbauer FH , van der Harst JJ , Groothoff JW , Coenraads PJ . Skin protection in nursing work: promoting the use of gloves and hand alcohol. Contact Dermatitis. 2004;51:135‐140. 15479202 10.1111/j.0105-1873.2004.00422.x

[75] Paula H , Hübner NO , Assadian O , et al. Effect of hand lotion on the effectiveness of hygienic hand antisepsis: Implications for practicing hand hygiene. Am J Infect Control. 2017;45:835‐838. 28768592 10.1016/j.ajic.2017.05.020

[76] Bundesinstitut für Risikobewertung . Neue Studien zu aluminiumhaltigen Antitranspirantien: Gesundheitliche Beeinträchtigungen durch Aluminium Aufnahme über die Haut sind unwahrscheinlich. Stellungnahme Nr. 030/2020 des BfR vom 20.07.2020; Available from: https://www.bfr.bund.de/cm/343/neue‐studien‐zu‐aluminiumhaltigen‐antitranspirantien‐gesundheitliche‐beeintr%C3%A4chtigungen‐durch‐aluminium‐aufnahme‐ueber‐die‐haut‐sind‐unwahrscheinlich.pdf [Last accessed June 26, 2024].

[77] Letzel M , Drexler H , Göen T , Hiller J . Impact of Daily Antiperspirant Use on the Systemic Aluminum Exposure: An Experimental Intervention Study. Skin Pharmacol Physiol. 2020;33:1‐8. 31553995 10.1159/000502239

[78] Diembeck W , Beck H , Benech‐Kieffer F , et al. Test guidelines for in vitro assessment of dermal absorption and percutaneous penetration of cosmetic ingredients. European Cosmetic, Toiletry and Perfumery Association. Food Chem Toxicol. 1999;37:191‐205. 10227743 10.1016/s0278-6915(98)00114-8

[79] Lademann J , Otberg N , Jacobi U , et al. Follicular penetration and targeting. J Investig Dermatol Symp Proc. 2005;10:301‐303. 10.1111/j.1087-0024.2005.10121.x 16382687

[80] Fischer T , Rystedt I . Skin protection against ionized cobalt and sodium lauryl sulphate with barrier creams. Contact Dermatitis. 1983;9:125‐130. 6221864 10.1111/j.1600-0536.1983.tb04318.x

[81] Korinth G , Geh S , Schaller KH , Drexler H . In vitro evaluation of the efficacy of skin barrier creams and protective gloves on percutaneous absorption of industrial solvents. Int Arch Occup Environ Health. 2003;76:382‐386. 12739171 10.1007/s00420-002-0429-y

[82] Korinth G , Weiss T , Penkert S , et al. Percutaneous absorption of aromatic amines in rubber industry workers: impact of impaired skin and skin barrier creams. Occup Environ Med. 2007;64:366‐372. 17182646 10.1136/oem.2006.027755 PMC2078516

[83] Korinth G , Lüersen L , Schaller KH , et al. Enhancement of percutaneous penetration of aniline and o‐toluidine in vitro using skin barrier creams. Toxicol In Vitro. 2008;22:812‐818. 18155878 10.1016/j.tiv.2007.11.006

[84] Korinth G , Wellner T , Luersen L , et al. Beschleunigung der dermalen Penetration von Kühlschmierstoffen und kanzerogenen Arbeitsstoffen durch Hautschutzcremes. Arbeitsmed Sozialmed Umweltmed 2006;41:106.

[85] Lodén M . The effect of 4 barrier creams on the absorption of water, benzene, and formaldehyde into excised human skin. Contact Dermatitis. 1986;14:292‐296. 3743039 10.1111/j.1600-0536.1986.tb05279.x

[86] Hui X , Maibach H . In vitro human skin percutaneous penetration: does a second topical application effect flux of first application? J Dermatolog Treat. 2022;33:916‐921. 32633593 10.1080/09546634.2020.1789539

[87] van der Bijl P , Gareis A , Lee H , et al. Effects of two barrier creams on the diffusion of benzo[a]pyrene across human skin. Sadj. 2002;57:49‐52. 11921637

[88] Robert Koch Institut . KRINKO: Indikationsgerechter Einsatz medizinischer Einmalhandschuhe | Ornithose‐Fälle in Deutschland. 26 June 2024; Available from: https://www.rki.de/DE/Content/Infekt/EpidBull/Archiv/2024/Ausgaben/10_24.pdf?__blob=publicationFile [Last accessed June 26, 2024].

